# Development of Parkinsonism following exposure to aripiprazole: two case reports

**DOI:** 10.1186/1752-1947-3-6448

**Published:** 2009-03-10

**Authors:** Lannah L Lua, Lin Zhang

**Affiliations:** 1 Department of Neurology, University of California Davis School of Medicine, 4860 Y Street, Suite 3700, Sacramento, California 95817, United States

## Abstract

**Introduction:**

Aripiprazole is a novel atypical neuroleptic used in the treatment of psychosis. A few recent studies have demonstrated an association between the use of aripiprazole and an exacerbation of Parkinsonism, although this relationship is poorly defined. To our knowledge, this is the first case series describing an onset of Parkinsonism in patients without prior history of Parkinson's disease following aripiprazole treatment.

**Case presentation:**

We describe two patients, ages 69 and 58, who developed cardinal features of Parkinson's disease shortly after receiving aripiprazole. Both patients were male veterans with a history of bipolar disorder treated with aripiprazole. They initially presented with asymmetric arm tremor, and subsequently developed rigidity, bradykinesia, and postural instability. On examination, they were found to be at a Hoehn and Yahr stage of 2.5 for their Parkinsonism.

**Conclusions:**

While aripiprazole has been associated with infrequent extrapyramidal side effects, these cases raise concerns that its chronic exposure may lead to D2 receptor hypersensitivity and/or dysfunction and subsequent development of a syndrome mimicking idiopathic Parkinson's disease. With the available atypical neuroleptics becoming widely used in treating psychotic symptoms associated with a broad range of disorders, we advise closer monitoring due to their potential for inducing Parkinsonism.

## Introduction

Aripiprazole is one of the recently introduced atypical antipsychotics (AAs) used in the treatment of psychosis related to schizophrenia [1], depression [2], bipolar disorder [3], and Parkinson's disease (PD) [4]. Well-documented side effects associated with the use of aripiprazole include insomnia, anxiety, headaches, nausea, vomiting, and somnolence [5]. As with other AAs, aripiprazole has been associated with fewer extrapyramidal side effects (EPS) that are thought to arise from a greater than 80% D2 receptor occupancy rate in the striatal area of the basal ganglia [6]. The differential EPS profile of aripiprazole can be attributed to its unique mechanism of action as a partial agonist at dopamine D2 and serotonin 5-HT1a receptors. Additionally, the drug functions as an antagonist at serotonin 5-HT2a receptors. Such mechanism of action is believed to stimulate dopamine release via a feedback loop in the basal ganglia [6]. Given its unique pharmacologic profile, aripiprazole may have a greater potential for worsening Parkinsonism than other AAs.

Many studies have extensively described the EPS profiles of earlier atypical neuroleptics, namely clozapine, quetiapine, olanzapine, ziprasidone and risperidone [6]. The relationship between aripiprazole treatment and Parkinsonism, however, remains poorly defined. Findings from recent studies have been inconsistent, reporting a wide range of motor effects following treatment with the agent [7]. Furthermore, the majority of these studies involve patients already diagnosed with PD. Given our limited experience with aripiprazole, its association with features of PD has yet to be delineated. In this report, we describe two unique cases of the association between aripiprazole treatment and the onset of Parkinsonism in patients without a prior history of PD or tremor.

## Case presentation

### Case report 1

Patient 1 is a 69-year-old, right-handed Caucasian male with a history of bipolar disorder type one. During the two years prior to his initial presentation in our movement disorders clinic, the patient had been taking aripiprazole daily for treatment of psychosis. Eighteen months following initial treatment, he developed right arm resting tremor and was subsequently given a higher dose of aripiprazole (10 mg) by his psychiatrist. Initially, his tremor occurred only at rest; later, he developed both resting and postural tremor, affecting his ability to hold things. His writing and other fine motor movements, however, were unaffected. Although no change in speech, cognition, or behavior had been noted, the patient reported that, in the months preceding his initial neurological evaluation, he had been unsteady on his feet, with an increased tendency to stumble. In spite of these symptoms, he remained independent and fully engaged in his activities of daily living.

On clinical examination, he was found to have mild hypomimia (see additional file 1) and hypophonia, and was also mildly bradyphrenic. He had resting and postural tremors, as well as mild rigidity of his arms, with symptoms being more prominent on the right (see additional file 1). He was also dysdiadochokinetic while seated and had a reduced arm swing when walking (see additional file 1). He scored 2.5 on the Hoehn and Yahr staging and 29/30 on the mini-mental status exam. The rest of his neurological examination was unremarkable, except for a reduced sensation to light touch and vibration in his distal lower extremities. His brain MRI scans were normal, with some age-appropriate volume loss. Due to fluctuations in his psychotic symptoms, aripiprazole could not be stopped or decreased. Sinemet was subsequently started, with noticeable improvement in tremors and gait. We observed further improvement in his Parkinsonism after the addition of trihexyphenidyl.

### Case report 2

Patient 2 is a 58-year-old, right-handed Caucasian male with a four-year history of bipolar disorder and post-traumatic stress disorder, for which he was placed on aripiprazole and depakote, respectively. While on both medications, he experienced a gradual onset of arm tremor on the right side, followed by the left side a few months later. Since this initial onset, his tremor progressed to a constant condition, occurring both at rest and while eating and drinking. Depakote was stopped a few months prior to his neurological evaluation, with no noticeable improvement in his tremor. In contrast, his tremors over the following months became more pronounced, affecting his drinking, eating, and writing. At this point, the patient also complained about gait unsteadiness leading to frequent falls.

On examination, the patient was hypomimic and mildly bradyphrenic. He had positive Myerson's sign, as well as rest and action/postural tremor. He also presented with rigidity of his arms, which was worse on the left, and an unstable, leftward leaning gait. His arm swing was reduced bilaterally, although worse on the left, and his arm tremors became more pronounced during ambulation. In addition, the patient was retropulsive. His Hoehn and Yahr staging was 2.5. He also demonstrated a decreased sensation to pinprick and vibration in the ankles. Overall, his symptoms were more prominent on the left side than on the right. As in the previous case, aripiprazole could not be stopped or decreased, but his tremors improved after two weeks on Sinemet.

## Discussion

The introduction of second-generation antipsychotics (SGAs) in the mid 1990s allowed clinicians to treat patients suffering from psychosis with a significantly reduced risk of developing EPS, compared to their first-generation counterparts, such as haloperidol [8]. The two cases presented in this report suggest that aripiprazole might be associated with the appearance of Parkinsonism when patients are on the drug for a certain length of time. While the optimal duration of exposure is currently not known for the induction of Parkinsonism [9], long-term use of these drugs should be monitored closely to avoid such adverse effects.

Although aripiprazole has been reported as one of the least vulnerable AAs in developing EPS based on its partial D2 agonistic and serotonin 5-HT1a agonistic property [4], the cases presented here raise concerns that its chronic exposure may lead to D2 receptor hypersensitivity and/or dysfunction and subsequent development of a parkinsonian syndrome mimicking idiopathic PD [10]. Thus, given its propensity to not only worsen, but also induce Parkinsonism, this medication should be administered with caution.

While both patients developed cardinal features of PD following treatment with aripiprazole, we are not certain whether these findings represent a drug effect or simply an unmasking of PD. Despite uncertainty regarding the actual mechanism, these reports further contribute to the literature demonstrating an association between exposure to aripiprazole and the onset of Parkinsonism. Our preliminary findings can serve as a starting point for future work that investigates the potential mechanism of action of aripiprazole leading to these unwanted effects.

With the increasing utilization of AAs in treating psychoses and psychotic symptoms in PD and other neurodegenerative disorders, we recommend close monitoring of these agents for safeguarding. A more comprehensive understanding of drug-receptor interactions of AAs may aid in a more efficacious utilization of these medications while minimizing the risk of developing and/or worsening Parkinsonism.

## Conclusion

We report two cases describing the association between the use of aripiprazole and the onset of Parkinsonism. This represents the first series of such an association in cases of psychosis without PD. Our study emphasizes the need for careful surveillance for the development of Parkinsonian features in patients treated with aripiprazole. The side effect profile of this medication has yet to be better characterized. Given the increasing clinical application of aripiprazole and other SGAs, we believe that further investigation on this relationship is warranted.

## Abbreviations

AAs: atypical antipsychotics; EPS: Extrapyramidal side effects; MRI: Magnetic resonance imaging; PD: Parkinson's disease; SGAs: Second-generation antipsychotics.

## Consent

Written informed consent was obtained from both patients for publication of these case reports. A copy of the written consent is available for review by the Editor-in-Chief of this journal.

## Competing interests

The authors declare that they have no competing interests.

## Authors' contributions

LL performed the background research and writing of the first and subsequent drafts. LZ was the treating clinician and was involved in the conception of the research project and the revision of the manuscript. LL and LZ were equally involved in the organization and execution of the research project, as well as in the review and critique of the manuscript. Both authors read and approved the final manuscript.

## 

## Supplementary Material

Additional file 1 Click here for file
